# Establishment of an in vitro thrombogenicity test system with cyclic olefin copolymer substrate for endothelial layer formation

**DOI:** 10.1557/s43579-021-00072-6

**Published:** 2021-09-07

**Authors:** Skadi Lau, Yue Liu, Anna Maier, Steffen Braune, Manfred Gossen, Axel T. Neffe, Andreas Lendlein

**Affiliations:** 1 Institute of Active Polymers and Berlin-Brandenburg Center for Regenerative Therapies, Helmholtz-Zentrum Hereon, Teltow, Germany; 2 Institute of Chemistry, University of Potsdam, Potsdam, Germany

**Keywords:** Biomedical, Chemical composition, Polymer

## Abstract

In vitro thrombogenicity test systems require co-cultivation of endothelial cells and platelets under blood flow-like conditions. Here, a commercially available perfusion system is explored using plasma-treated cyclic olefin copolymer (COC) as a substrate for the endothelial cell layer. COC was characterized prior to endothelialization and co-cultivation with platelets under static or flow conditions. COC exhibits a low roughness and a moderate hydrophilicity. Flow promoted endothelial cell growth and prevented platelet adherence. These findings show the suitability of COC as substrate and the importance of blood flow-like conditions for the assessment of the thrombogenic risk of drugs or cardiovascular implant materials.

**Graphic abstract:**

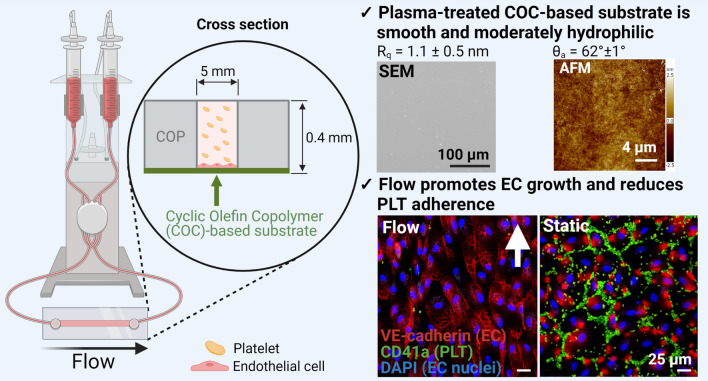

**Supplementary Information:**

The online version contains supplementary material available at 10.1557/s43579-021-00072-6.

## Introduction

Thrombotic events are one of the major risks of new medicinal products and associated excipients. An up-to-date example for this is the occurrence of some severe cases of thrombotic complications in cerebral and splanchnic veins upon vaccination with ChAdOX1 nCov-19 (AstraZeneca) to provide protection against the SARS-CoV-2-induced pandemic causing Corona Virus Disease 2019 (Covid-19).^[1]^ Though no changes in coagulation were detected in pre-clinical and clinical trials with the AstraZeneca vaccine and though a causal link is not proven,^[2]^ this scenario demonstrates once more the overall need to investigate the effect of new medicinal products such as vaccines and associated excipients as detailed as possible, preferably as early as in preclinical in-vitro studies. Ideally, as drugs ultimately get into contact with the vasculature, a blood vessel-like situation including endothelial cells (EC), platelets (PLT) and blood flow-like hydrodynamic conditions should be implemented in such a test system. Commercially available perfusion systems are equipped with polymer-based slides as substrates for EC. Besides conferring EC adhesion and supporting the formation of a confluent cell layer, these substrates must provide optical properties to allow microscopic cell monitoring over the duration of the experiment. Cyclic olefin copolymers (COC) fulfill these optical requirements and are thus increasingly applied in microfluidic systems.^[3]^ Generally, COC are known as packaging material for food and as a material for syringes, blisters and vials in the pharmaceutical sector.^[4]^ Recently, COC was even discussed to be a potential new implant material due to its biocompatibility.^[5]^ A major advantage of using COC is that its surface properties can be varied by chemical modification such as plasma treatment, which is a prerequisite for EC adhesion.^[6]^

Here, composite slides composed of a cyclic olefin polymer (COP)-based upper part and a COC-based lower part were used. The latter was surface modified by plasma treatment and functioned as a substrate for EC growth. This plasma-treated COC-based substrate was comprehensively examined regarding physicochemical properties aiming at the establishment of a near-physiological in vitro test system suitable to investigate the thrombogenic risk of new medicinal products such as vaccines. Moreover, first co-culture studies were performed using plasma-treated COC as a substrate to investigate the EC-PLT interplay on this material, both under static conditions or unidirectional laminar flow.

## Materials and methods

### Characterization of COC-based bulk material

Optical properties such as transmission was recorded in the UV/visible light range using a spectrophotometer (Cary 50 Bio, Varian Inc. Agilent Technologies, Santa Clara, USA) at 25°C. The refractive index was measured using sunlight with an Abbe refractometer (Carl Zeiss, Jena, Germany) at 25°C. To investigate the chemical structure and cyclic monomer content of COC-based cell substrates (Coverslips for sticky-Slides, Polymer coverslip, Ibidi, Gräfelfing, Germany), ^1^H and ^13^C Nuclear Magnetic Resonance (^1^H NMR and ^13^C NMR) spectra were recorded at 68°C using toluene-d8 as a solvent on a Bruker Ascend 700 spectrometer (700 MHz for ^1^H and 175 MHz for ^13^C, Bruker, Karlsruhe, Germany). Functional groups were further examined by Attenuated total reflection Fourier transform infrared (ATR-FTIR) spectroscopic analysis on an Avatar 360 (Thermo Nicolet, USA) over a range of 500–4000 cm^−1^ at a resolution of 8 cm^−1^. Weight and number average molar masses (*M*_w_, *M*_n_) and polydispersity of the polymer were determined by high temperature multidetector gel permeation chromatography (GPC), performed by PSS Polymer Standard Service GmbH. The GPC system consisted of two PSS POLEFIN columns (ID 8.0 mm × 200.0 mm, Polymer Standards Service GmbH, Mainz, Germany), an isocratic pump (Agilent, Waldbrunn, Germany), an automatic injector (PolymerChar, Valencia, Spain), an IR4 detector (PolymerChar, Valencia, Spain), and a viscosity detector (Polymer Standards Service GmbH, Mainz, Germany). Samples were dissolved in 1,2,4-Trichlorobenzene at 160°C for two hours with a concentration of 3 mg mL^−1^ and the measurements were performed at 160°C. Samples were universally calibrated with polystyrene standards using a viscometer (Polymer Standards Service GmbH, Mainz, Germany). GPC data were evaluated using WinGPC unichrom v.8.33 (Polymer Standards Service GmbH, Mainz, Germany). Differential Scanning Calorimetry (DSC) experiments were conducted on a Netzsch DSC 204 Phoenix (Selb, Germany) at heating and cooling rates of 10 K min^−1^ in sealed aluminum pans. In particular, the polymer samples were submitted to two testing cycles starting with a heating phase from room temperature to 200°C before they were cooled down to − 50°C and reheated to 200°C. The glass transition temperature (*T*_g_) was determined from the second heating run. Thermo gravimetric analyses (TGA) were conducted on a thermo microbalance (Netzsch GmbH TG 209C) under a nitrogen atmosphere within the temperature range of 25–800°C at a heating rate of 10 K min^–1^. Wide angle X-ray scattering (WAXS) measurement was carried out at ambient temperature on a D8 Discover diffractometer (Bruker AXS, Kalsruhe, Germany) with Cu *K*_α_ radiation (λ = 0.154 nm) at a voltage of 40 kV and a current of 40 mA. The scattered X-rays were registered with a 2D-detector with a resolution of 100 µm. The distance between sample and detector was 15 µm. The recorded 2D diffraction data were subjected to standard data correction and evaluation procedures (using TOPAS® software of Bruker AXS, Karlsruhe, Germany) to obtain 1D scattering curves.

### Surface characterization of cyclic olefin copolymer film

Scanning electron microscopy for characterizing the COC substrate surface topography as well as a cross-section of the channels were conducted at room temperature using an environmental scanning electron microscope (ESEM; Quanta 250 FEG, FEI Deutschland, Frankfurt am Main, Germany). The samples were coated with a 4 nm iridium layer (QT-Target-Iridium-3, Lot Quantum Design GmbH, Germany), scanned at high vacuum with 5 kV and analyzed with an Everhart–Thornley detector (secondary electrons). To image the cross-section of the channel, slides were cut by a saw and a scalpel prior to ultra microtome cutting (EM UC 6, Leica, Wetzlar, Germany) using glass knifes (EM KMR 2, Leica, Wetzlar, Germany) at room temperature. The surface topography and nano-roughness of COC films was further examined by atomic force microscopy (AFM) on a MFP-3D (Asylum Research, Santa Barbara, CA, USA). A silicon cantilever (OLYMPUS OMCL AC160TS-R3), with a driving frequency of around 150 kHz and a spring constant of 9 N m^−1^ was utilized at typical scan rates of 0.5 Hz. Five different areas with a scan size of 5 × 5, 20 × 20, 50 × 50 μm^2^ were investigated. Water contact angle (CA) measurements were carried out using a drop shape analyzer (DSA 100, Krüss GmbH, Hamburg, Germany). For the measurements, ultra-pure deionized water provided by an Ultra Clear UV clean water system (SG Wasseraufbereitung und Regenerierstation GmbH, Barsbüttel, Germany) with a conductivity of 0.055 μS cm^−1^ was used. The sessile drop method was used to characterize the samples at room temperature.

### Isolation of platelet-rich plasma (PRP) and platelet-poor plasma (PPP) from whole blood

Whole blood anticoagulated with trisodium citrate (German Red Cross, Berlin, Germany) of 3 apparently healthy donors (1 male/2 female; 30–64 years resp. 51 ± 18 years) was used in accordance with the ethics committee of Charité University Medicine Berlin (EA2/012/10). To exclude blood anomalies, the blood was subjected to a quality test according to the guidelines of the Nordkem workshop.^[7, 8]^ While platelet-rich plasma (PRP) was isolated by centrifugation of whole blood at 140×*g* for 20 min (Centrifuge 5804 R, Heraeus, Hanau, Germany), platelet-poor plasma (PPP) was obtained by centrifugation at 1500×*g* for 30 min at 22°C and used to adjust the target concentration of PRP.

### Co-culture of endothelial cells and platelets

Human umbilical vein endothelial cells (HUVEC, Passage 6, Lonza, Cologne, Germany) were seeded in plasma-treated µ-slides I Luer^0.4^ (Ibidi, Gräfelfing, Germany) with a density of 1.2 × 10^6^ mL^−1^ and cultivated in EGM-2 medium (Lonza, Cologne, Germany) in a standard humidified incubator at 37°C with 5% (v/v) CO_2_. After 24 h, HUVEC were exposed to laminar flow (10 dyn cm^−2^) using a commercial pump system (Ibidi, Gräfelfing, Germany). In parallel, endothelialized slides used for quasi-static co-culture experiments were connected to a syringe pump that ensured an extremely slow medium flow (0.013 mL min^−1^) resulting in an equally low shear stress of 0.01 dyn cm^−2^. After six days, 300,000 PLT µL^−1^ were added in a 1:1 ratio to the EGM-2 medium resulting in a final PLT concentration of 150,000 PLT µL^−1^. After 1 h, the number of adherent HUVEC and PLT was quantified upon immunostaining and microscopy (see below) followed by image analysis using ImageJ (version 1.48c, Java 1.8.0-261).

### Immunostaining

EC and PLT were washed with phosphate buffered saline (PBS, Gibco, Paisley, United Kingdom) and fixed with 4% (w/v) paraformaldehyde (Sigma, Steinheim, Germany) for 30 min at room temperature. Blocking of unspecific binding sites was performed with 5% (w/v) bovine serum albumin (BSA, Roth, Karlsruhe, Germany) dissolved in PBS for 30 min. EC were visualized by mouse anti-human VE-cadherin primary antibodies (Santa Cruz Biotechnology, Heidelberg, Germany, sc-9989, 1:500) and secondary donkey anti-mouse IgG antibodies conjugated to Alexa Fluor® 555 (Thermo Fisher Scientific, Hennigsdorf, Germany, A31570, 1:40). PLT were labelled with mouse anti-human CD41a antibodies conjugated to FITC (Miltenyi Biotec, 130-120-719, 1:50). Antibodies were diluted in PBS containing 2% (w/v) BSA and 0.3% (v/v) Triton-X-100 (Roth, Karlsruhe, Germany). After 1 h of incubation in the dark, slides were washed with PBS and endothelialized slides were stained with DAPI (Roth, Karlsruhe, Germany, 1:50) for 5 min to counterstain cell nuclei. Eventually, slides were washed and mounted with mowiol (Roth, Karlsruhe, Germany). Microscopic images were taken using a light microscope (Axiovert 40, Zeiss, Jena, Germany) and a confocal laser scanning microscope (DMi8 TCS SP8, Leica Microsystems GmbH) at 20-fold primary magnification. Phase contrast images were taken to manually count EC before and after co-culture with PLT and to quantify the alignment of EC towards the direction of flow described by the Feret Angle and measured by ImageJ (*n* = 30; three independent experiments with two slides per donor and five images per slide). Fluorescent images were taken to evaluate the number and area of adhered PLT using ImageJ (non-endothelialized slides: *n* = 30; three independent experiments with two slides per donor and five images per slide; endothelialized slides: *n* = 20; three independent experiments with two slides per donor and four images per slide).

### Statistics

Statistical analyses were performed using Graphpad Prism 6 (Graphpad Software, San Diego, California). Gaussian distribution of the data was tested using the Kolmogorov–Smirnov test. Comparisons of non-parametric data between multiple groups were performed by Kruskal–Wallis test and Dunn’s posttest. Differences were considered significant at *p* ≤ 0.05.

## Results and discussion

**Figure 1:**
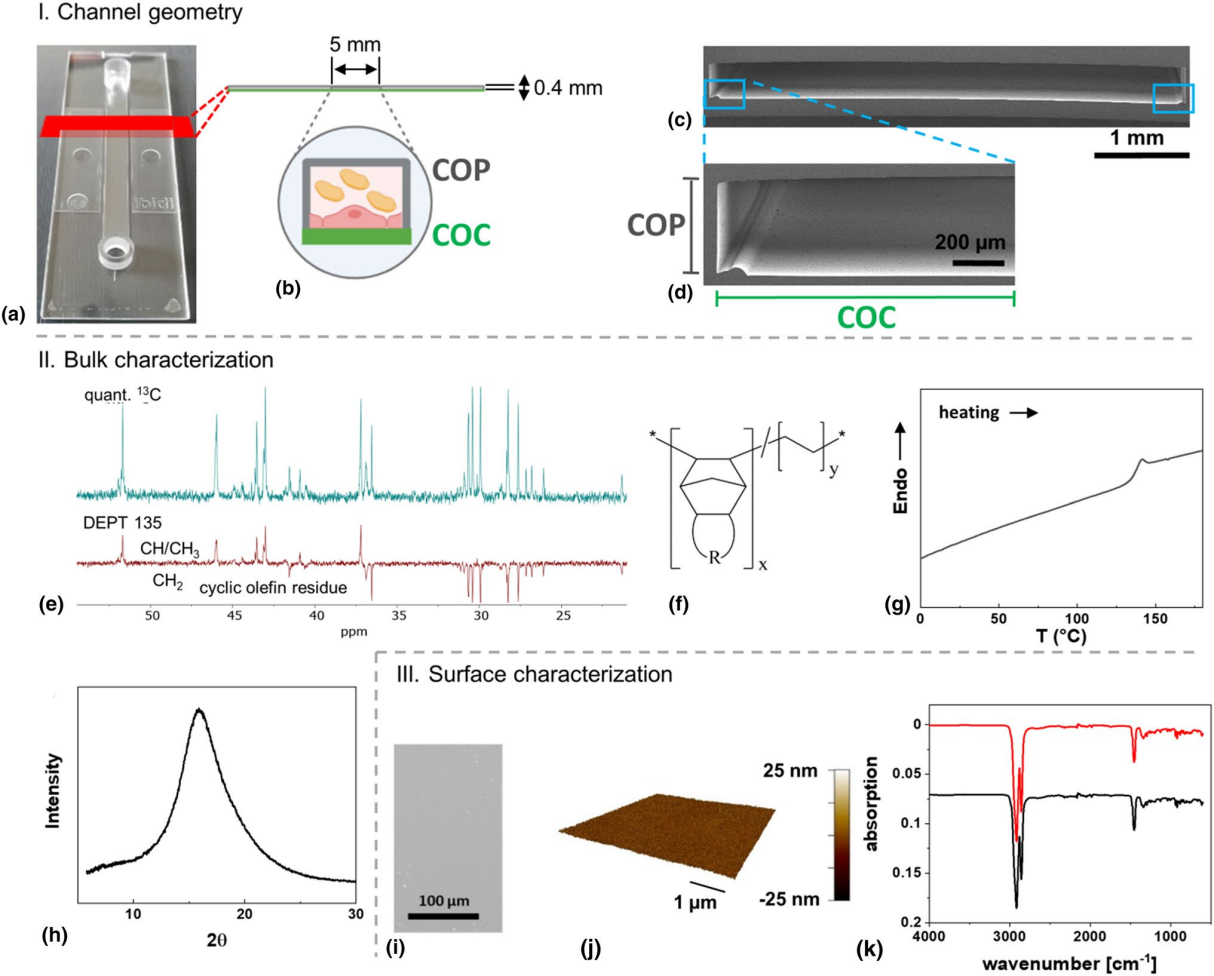
Physicochemical characterization of the cyclic olefin copolymer (COC)-based substrate. (I) Channel geometry: (a) µ-slide I Luer^0.4^; (b) schematic image of the µ-slide I Luer^0.4^ consisting of an upper cyclic olefin polymer (COP)-based part and a lower plasma-treated COC-based part functioning as a substrate for endothelial cells; (c) ESEM image of the cross section of the channel; (d) magnification of the left side of the channel showing the COP-based channel walls and the COC-based substrate used for cell adhesion. (II) Bulk characterization: (e) ^13^C NMR spectra of COC (top: inverse gated decoupling ^13^C with a d1 = 25 s, bottom: DEPT 135) and predicted chemical structure (f); (g) DSC curve and (h) WAXS curve of COC sample. III. Surface characterization: (i) ESEM image and (j) AFM height image of plasma-treated COC-based substrate surface; (k) ATR-FTIR spectrum of the plasma-treated (red) and non-treated (black) COC.

The perfusion system used for the establishment of a thrombogenicity test setup allows the use of standardized slides as substrate for endothelial cells. Here, we explored COC-based surface modified films for their suitability in the test system. The slide exhibits a rectangular shape with an integrated rectangular channel used for cell adhesion [Fig. 1(a, b)]. ESEM analysis of the cross section of the channel revealed that the COC-based substrate at the bottom of the slide is not entirely planar but exhibits a bulge-like structure at the channel borders. This most likely resulted from the production process used to connect the upper cyclic olefin polymer (COP)-based part defining the channel and the COC-based film on the bottom [Fig. 1(c, d)]. As substrate geometry is known to influence endothelial cell behavior,^[9]^ it cannot be entirely excluded that cells at these positions affected the remaining cells. For example, Schulz et al. demonstrated that non-adherent cells can influence the morphology and function of adherent endothelial cells and thus the assessment of the endothelialization capacity of implant materials.^[10]^

In the following, optical properties such as transmission and refractive index of COC, measured by a spectrophotometer and an abbe refractometer, are described. Moreover, the chemical structure, polymer morphology, and thermal behavior of the COC-based substrate, which were analyzed by NMR, GPC, WAXS, DSC, and TGA measurements, are described. The surface properties were mainly determined by ESEM, AFM and CA experiments.

COC used in the present study exhibits a refractive index of 1.53, which is similar to the refractive index of glass. The transparency of COC for visible light is 92% above 350 nm wavelength, which is also similar to glass. Moreover, it is known that COC permits UV light transmission while other common substrate materials such as glass or polystyrene block UV light.^[11]^

The ^1^H NMR (Figure S1) and ^13^C NMR [Fig. 1(e)] spectra show signals for a typical hydrocarbon, which is confirmed in the FT-IR spectrum [Fig. 1(k)] that does not show any bands typically associated with heteroatoms such as oxygen, nitrogen or halogens. Because of the peak separation in the ^13^C together with the DEPT 135 spectrum differentiating between CH/CH_3_ and CH_2_ groups, the ^13^C allows for partial structural assignment. The acquired spectra are not conform with published spectra of COCs but have certain similarities with COCs based on multicyclic cyclic olefin components such as tetracyclododecene.^[12]^ The peak at ~ 51.7 ppm is especially noteworthy, as most COCs show only peaks < 50 ppm.^[12]^ The relatively high shift is consistent with a bridgehead tertiary carbon in a multicyclic structure [Fig. 1(f)]. Spectra of COC may show a complex signal structure due to microheterogeneity stemming from various sequence structures, stereo- and regioisomers as well as endo/exo diastereomers in multicyclic structures.^[13]^ Assuming, consistent with spectra from assigned COC, that the peaks with a chemical shift below 30.7 ppm are related to the ethylene unit and two secondary carbons from the cyclic olefin unit, a rough estimation of the cyclolefin molar content can be calculated to be ~ 24 mol%. In addition, most of the ^13^C signals are well separated, which putatively indicates a cyclolefin content below 50 mol%, based on the example of COC containing norbornene units.^[14]^ GPC analysis revealed a number average molar mass *M*_n_ = 14.4 ± 0.4 kg mol^−1^ of COC and a polydispersity index *M*_w_/*M*_n_ of 1.98. Compared with the other commercial cyclic olefin copolymers,^[15]^ it has to be pointed out that the values given here were determined from universally calibrated GPC, while the literature values were determined by standard calibration,^[15]^ which may also be the reason for the differences.

The thermal properties were examined by DSC and TGA analysis. The glass transition temperature (*T*_g_) determined by DSC is 137 ± 2°C [Fig. 1(g)], which is in the range of the commercially available COC. The *T*_g_ of COCs increases with the cycloolefin content, as has been shown for several COCs.^[16]^ The *T*_g_ generally increases with decreased mobility of chain segments or individual groups, and has been shown to be higher for COCs containing multicyclic structures compared to the norbornene-based COC, comparing COC with the same molar content of the cycloolefin. For example, a tetracyclododecene-based COC with 28 mol% cycloolefin shows a *T*_g_ of 125°C, while the comparable COC based on norbornene has a *T*_g_ of < 80°C.^[17, 18]^ Aligning these observations regarding the *T*_g_ of COCs with the NMR results, the DSC further confirms our interpretation that the here investigated COC is based on a multicyclic cycloolefin with an estimated molar content of ~ 24 mol%.The degradation behavior was characterized by TGA (Figure S2) and revealed an onset degradation temperature (*T*_d_) of 445 ± 2°C, at which 10% weight loss occurred. The maximum degradation temperature (*T*_max_) is 473 ± 2°C, as evaluated by the peaks in relative derivative thermogravimetry (DTG) curves. The sample shows only one degradation step in the temperature interval of 445–490°C with 100% weight loss, which is probably attributed to both degradation of cyclic moiety and ethylene units. This copolymer exhibits a high thermal stability, similar to the other types of COC.^[17]^ COCs based on tricyclo[4.3.0.1^2,5^]deca‐3‐ene, tricyclo[4.4.0.1^2,5^]undec‐3‐ene, and tricyclo[6.4.0.1^9,12^]tridec‐10‐ene can be excluded from the list of potential candidates, as they show two or three steps of thermal degradation.^[18]^

WAXS measurement was performed to gain morphological information. One halo with a peak at 15.9° can be observed in the WAXS diffractogram [Fig. 1(h)]. This peak is sharper than an amorphous halo, but it should be emphasized that this is not a crystalline reflection, as it is already known that COCs are noncrystalline.^[14, 19]^ This phenomenon is comparable to the reported norbonene-based COC with one halo at 17°, which even developed a low-angle shoulder when increasing the norbonene content above 50 mol%.^[14]^

Based on the ESEM picture and AFM height image [Fig. 1(i, j)], it can be observed that the COC-based substrate surface is planar with a low surface roughness (*R*_q_ = 1.1 ± 0.5 nm). These results are comparable to other cell culture substrates.^[20]^ The COC-based substrate investigated here shows less needle- or globules-like structures on the surface, indicating a better homogeneity and reduced surface factor compared to an ethylene-norbornene copolymer assessed for biocompatibility.^[5]^ The water contact angle of untreated COC surfaces is 107 ± 1°, which is similar to other commercial, hydrophobic COCs.^[12]^ The water advancing contact angle of the plasma-treated COC surface is reduced to 62 ± 1°. This demonstrates that the plasma treatment rendered the COC surface more hydrophilic, which has been connected to enhanced cell attachment. This may be mediated either through direct surface attachment or through binding to adherent proteins although lowering of the contact angle also reduces the adsorption of proteins.^[21]^ Endothelial cell adhesion was indeed efficient, even under flow conditions. However, the adsorption of proteins is equally important as they can contribute to thrombosis formation. According to the Berg limit, unspecific protein adsorption is likely to occur above a critical water contact angle of 60–65°, while below this limit, unspecific protein adsorption is limited.^[22, 23]^ As the water contact angle of the COC is exactly in the range of the Berg limit, it remains to be investigated whether protein adsorption is promoted.

The surface chemistry of the plasma-treated and non-treated COC films was further studied with ATR-FTIR. Both spectra show absorption bands typical for hydrocarbons with bands at 2858 cm^−1^ and 2916 cm^−1^ indicating C–H stretching and a band at ~ 1455 cm^−1^ indicating alkane bending vibrations [Fig. 1(k)]. No obvious characteristic peak of carbonyl, aldehyde or hydroxyl groups are observed in case of the plasma-treated side. It has been reported that norbornene-based COC film surfaces (i.e. TOPAS®) presented small peaks in FTIR indicating carbonyl, aldehyde or hydroxyl groups right after plasma treatment, and had a contact angle increased from around 20° to 60° after ageing of 180 days.^[21]^ In that case the peak height and area for the hydroxyl group decreased upon ageing due to the restructuring and reorientation of the polar surface, but the FTIR-ATR peak corresponding to carbonyl groups increased with ageing due to post-plasma reactions between trapped active radicals and atmospheric oxygen. However, peak intensities were very low in these studies. We detected only negligible changes by ATR-FTIR when comparing treated and untreated surfaces, which may reflect the too low sensitivity of the method in view of the limited change of the chemical structure due to aging or the low extent of modification. Aging here induced structural rearrangements of chains due to relaxation effects, which may have been enhanced in the processing of the device when combining parts from different polymers. For future studies XPS may be a method of choice to detect changes of chemical groups with high resolution on the surface of the treated COC.

**Figure 2:**
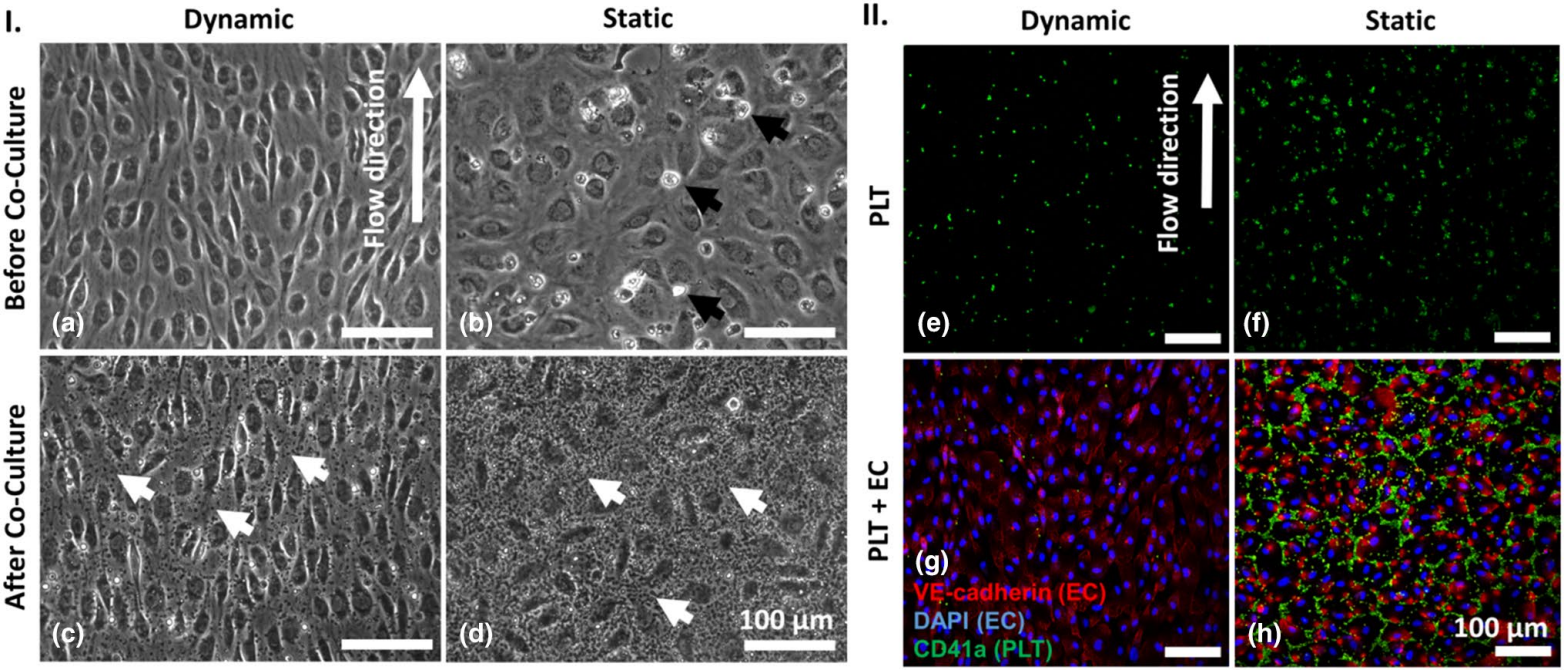
Endothelial cells (EC), platelets (PLT) and co-cultures of EC and PLT under dynamic and quasi-static conditions. (I) Phase contrast images: EC after six days of cultivation under laminar flow (10 dyn cm^−2^, a) or quasi-static conditions (0.01 dyn cm^−2^, b) prior to co-culture (black arrows indicate dead EC); EC and PLT (visible here as small, granular speckles indicated by white arrows) after 1 h of co-culture under laminar flow (c) or quasi-static conditions (d). (II) Immunostaining: PLT (CD41a-FITC-labeled, green) cultivated in the absence of EC under laminar flow (e) or quasi-static conditions (f); Co-cultures of EC (VE-cadherin-Alexa Fluor 555- and DAPI-labeled, red with blue nuclei) and PLT after 1 h under laminar flow (g) or quasi-static conditions (h).

EC were cultivated for 6 days under static or dynamic conditions prior to the addition of PLT for 1 h. Six days of EC cultivation under dynamic conditions resulted in the formation of a dense monolayer of EC, which aligned in the direction of flow [Fig. 2(a)]. In contrast, under static conditions, fewer EC were present and those exhibited a cobblestone-like morphology. To quantify the alignment of EC under dynamic and static conditions, the Feret Angle, a measure to describe the cell alignment towards to direction of flow, was determined. While angles of 0° and 90° indicate a fully parallel or vertical alignment, respectively, an angle of 45° represents a random cell orientation.^[24]^ Here, EC cultivated under static conditions exhibited a Feret Angle of 42 ± 23°, thus displaying an almost completely random orientation. In contrast, EC cultivated under flow exhibited a Feret Angle of 25 ± 16° indicating that these cells aligned towards the direction of flow (Figure S3). Moreover, considerably more dead cells were observed compared to dynamically cultivated EC [Fig. 2(b), white cells/debris]. After co-culture, less PLT were detected upon laminar flow compared to static conditions [Fig. 2(c, d), small grey dots].

To investigate the effect of flow per se on PLT, they were initially cultivated in the absence of EC. Under this condition, less PLT adhered under laminar flow than under static conditions [Fig. 2(e, f)]. In a subsequent experiment, endothelialization of COC slightly reduced the number of adherent PLT under dynamic conditions [Fig. 2(g)]. In contrast, most PLT adhered to endothelialized slides under static conditions [Fig. 2(h)].

**Figure 3:**
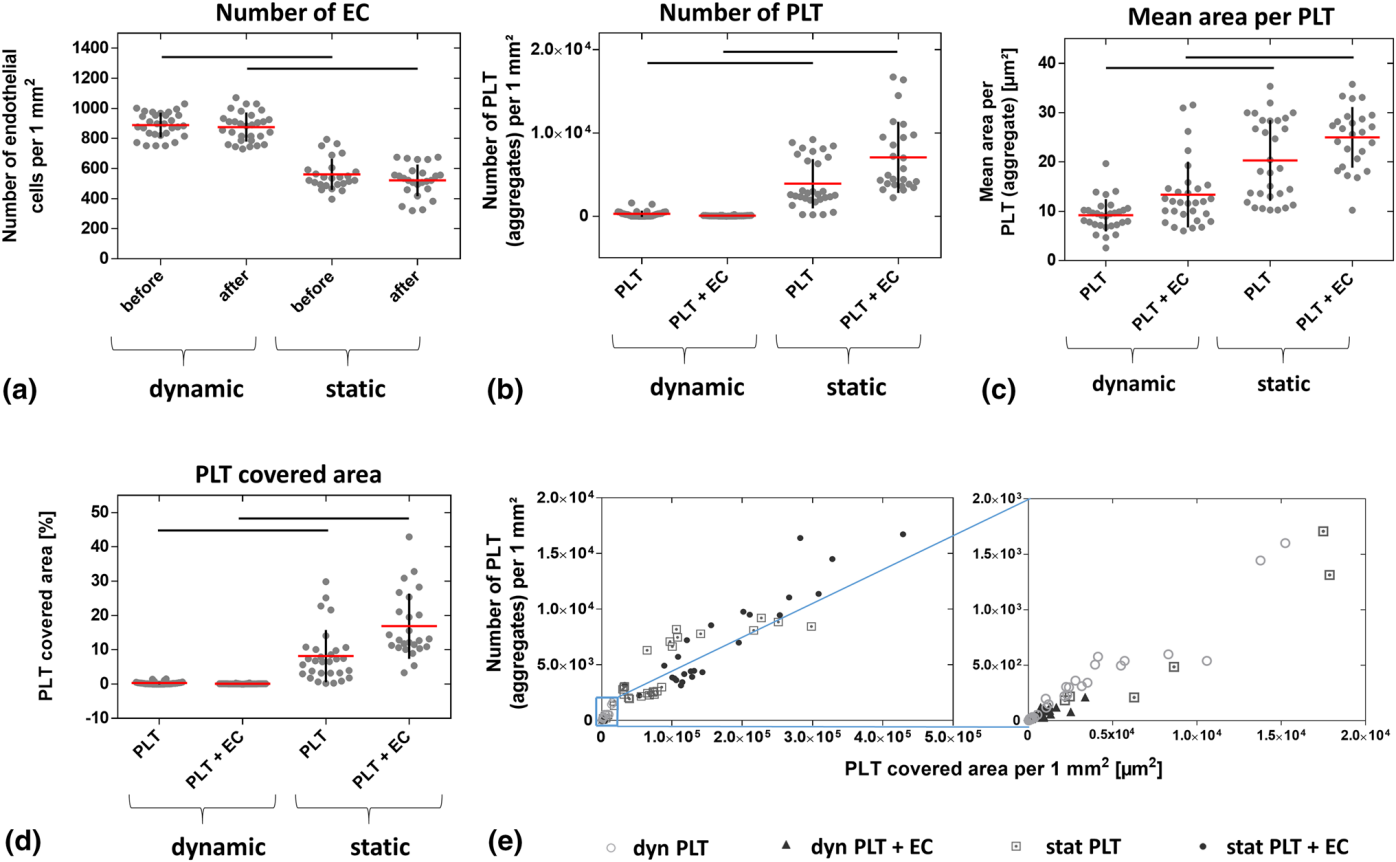
Number of adherent endothelial cells (EC) and platelets (PLT) as well as mean area per PLT and PLT covered area after co-culture under dynamic and quasi-static conditions. EC were cultured for six days either under laminar flow (10 dyn cm^−2^) or quasi-static conditions (0.01 dyn cm^−2^) prior to the addition of PLT. Shown are the number of EC (a) and PLT (b) as well as the mean area per PLT (c) and PLT covered area (d) before and after co-culture under dynamic and static conditions. Figure E shows the relation between PLT number and PLT covered area. Data in the blue rectangle of the left image are magnified in the right image. Arithmetic mean (red) ± standard deviation (black). Data are mean from three independent experiments using PLT of one donor per experiment cultivated on one to two slides whereas five images were taken per slide (*n* = 25–30). Lines = *p* < 0.0001. Legend: empty circle: PLT incubated under laminar flow, triangle: PLT incubated with EC under laminar flow, square with point: PLT incubated under static conditions, black circle: PLT incubated with EC under static conditions.

To evaluate these results in a quantitative manner, the number of adherent EC and PLT as well as mean area per PLT and PLT covered area were analyzed. Though equal numbers of EC were seeded at day 0, significantly more EC were present after cultivation under laminar flow (889 ± 82 EC per mm^2^) than under static conditions at day 6 (561 ± 104 EC per mm^2^, *p* < 0.0001). This indicates that flow is beneficial for EC growth under the conditions tested and it underlines the importance of blood flow-like conditions for in vitro studies using EC. After co-cultivation with PLT for 1 h, the number of EC was almost unchanged for both static (521 ± 105 EC per mm^2^) and dynamic conditions (876 ± 97 EC per mm^2^) [Fig. 3(a)]. This indicates that EC were not activated by PLT, which might have led to their detachment from the substrate.^[25]^

Determination of the PLT number, including single PLT and PLT aggregates, revealed that few PLT adhered to the substrate under laminar flow (PLT: 303 ± 389 PLT per mm^2^ and PLT + EC: 64 ± 44 PLT per mm^2^). In contrast, under static conditions, high numbers of PLT adhered to non-endothelialized COC (3910 ± 2957 PLT per mm^2^) and even more PLT adhered to endothelialized COC (7072 ± 4266 PLT per mm^2^), particularly to the inter-endothelial space. It is well known that PLT adherence is mediated by the subendothelial extracellular matrix, predominantly by collagen, von Willebrand factor and fibronectin among others.^[26]^ It therefore is likely that the pattern of adherent PLT around EC is due to the interaction with endothelial extracellular matrix components. However, statistically significant differences were only related to the type of cultivation (static versus dynamic, *p* < 0.0001; Fig. 3(b)], not to the presence of EC. To investigate whether PLT exclusively adhered to COC, the upper COP-based part of the µ-slide and different positions if the silicone tubings were investigated regarding platelet adherence in the scope of preliminary experiments. Under the conditions tested (1 h under flow) no notable PLT adherence was found at any of these positions. Thus, PLT adherence was restricted to COC.

The surface area per PLT (also including aggregates) was lowest when cultured under laminar flow (9.2 ± 3.3 µm^2^ for non-endothelialized COC). Similar to the PLT number, no significant difference was detected upon endothelialization of COC (13.4 ± 6.7 µm^2^), indicating that endothelialization does not further reduce the thrombogenicity of COC-based substrates. The highest mean areas per PLT were measured for PLT cultivated under static conditions with or without endothelialization (PLT: 20.3 ± 8.2 µm^2^ and PLT + EC: 25.0 ± 6.2 µm^2^) compared to dynamic conditions (PLT: 9.2 ± 3.3 µm^2^ and PLT + EC: 13.4 ± 6.7 µm^2^; *p* < 0.0001) [Fig. 3(c)].

A similar pattern was observed for the PLT covered area. While a minimal area was covered by PLT under dynamic conditions regardless of the presence of EC (PLT: 0.3 ± 0.4% and PLT + EC: 0.1 ± 0.1%), PLT covered a greater area under static conditions (PLT: 8.1 ± 7.6% and PLT + EC: 16.9 ± 9.5%). Again, statistically significant differences were limited to the type of cultivation (static versus dynamic, *p* < 0.0001), not to the presence of EC [Fig. 3(d)]. Figure 3(e) illustrates the relationship between the PLT number and PLT covered area, which are both increased under static conditions (left image) compared to laminar flow (magnification of left image section).

These data confirm the importance of cultivating EC and PLT under flow. Previous work has shown that laminar flow, especially at higher rates, can prevent the development of atherosclerosis while disturbed or oscillatory shear stress, as present at vessel bifurcations, creates an atheroprone environment (reviewed in^[27]^). Different thresholds for shear rates of 2600 s^−1^, 6000 s^−1^ and even 20,000 s^−1^ have been claimed after which PLT adherence decreased.^[28–30]^ Here, a comparably low shear rate of 1100 s^−1^ was applied, which is in the physiological range of small arteries (reviewed in^[31]^). Though this was much lower compared to the critical shear rates mentioned above, PLT adherence was moderate, even under static conditions. Nevertheless, as a certain increase in the shear rate is known to concentrate erythrocytes in the central layers of the blood flow thereby forcing PLT towards to vessel wall and thus increasing the risk of thrombus formation, future studies should also test higher shear rates using the test system applied here.^[31]^ Interestingly, endothelialization of COC-based substrates did not further reduce the adherence of PLT. For other materials such as PTFE, it is known that endothelialization considerably reduces PLT adherence.^[32]^ It can be speculated that the naturally low thrombogenic potential of COC is responsible for both the moderate PLT adherence and the nonexistent effect of EC on PLT adherence.^[5]^ Moreover, it has to be noted that the current data were collected using platelet-rich plasma, not whole blood, thereby avoiding additional donor-related variation in line with our goal of establishing a potentially standardized test system. However, as platelet adherence is known to increase with an increasing hematocrit (volume fraction of erythrocytes), it can be speculated that PLT adherence to COC in the presence of erythrocytes may be higher than shown here.^[33]^

## Conclusion

First fundamental steps were undertaken to establish a dynamic in vitro test system for the assessment of new substances such as drugs and excipients regarding their thrombogenic risk under near-physiological conditions. The investigated COC-based substrate proved suitable for endothelialization and the improved EC growth and reduced PLT adherence under flow underlines the importance of blood flow-like conditions for in vitro studies using these cells. Future studies will evaluate the test system using different substances and their effect on the interplay of EC and PLT. This could be performed with platelets from healthy blood donors or from patients having platelet-mediated disorders to extend the application spectrum of the thrombogenicty test system. Moreover, the test system could be extended to the assessment of cardiovascular implant materials.

## Supplementary Information

Below is the link to the electronic supplementary material.

Supplementary file1 (PDF 696 kb)

## Data Availability

The datasets generated during and/or analyzed during the current study are available from the corresponding author on reasonable request.
