# Supplementary material for: Establishment of an in vitro thrombogenicity test system with cyclic olefin copolymer substrate for endothelial layer formation
Source: MRS Commun. 2021 Sep 7;11(5):559–67. doi: 10.1557/s43579-021-00072-6 (PMC8422954; doi:10.1557/s43579-021-00072-6)
Supplement: Supplementary file 1 — Supplementary file1 (PDF 696 kb) [file 43579_2021_72_MOESM1_ESM.pdf]

Supporting Information for

In Vitro Thrombogenicity Test System with Cyclic Olefin Copolymer Substrate for Endothelial Layer Formation

Skadi Lau[1], Yue Liu[1], Anna Maier[1], Steffen Braune[1], Manfred Gossen[1], Axel T. Neffe[1], Andreas Lendlein[1,2,*]

1: Institute of Active Polymers and Berlin-Brandenburg Center for Regenerative Therapies, Helmholtz-Zentrum Hereon, Teltow, Germany

2: Institute of Chemistry, University of Potsdam, Potsdam, Germany

*Corresponding author: andreas.lendlein@hereon.de

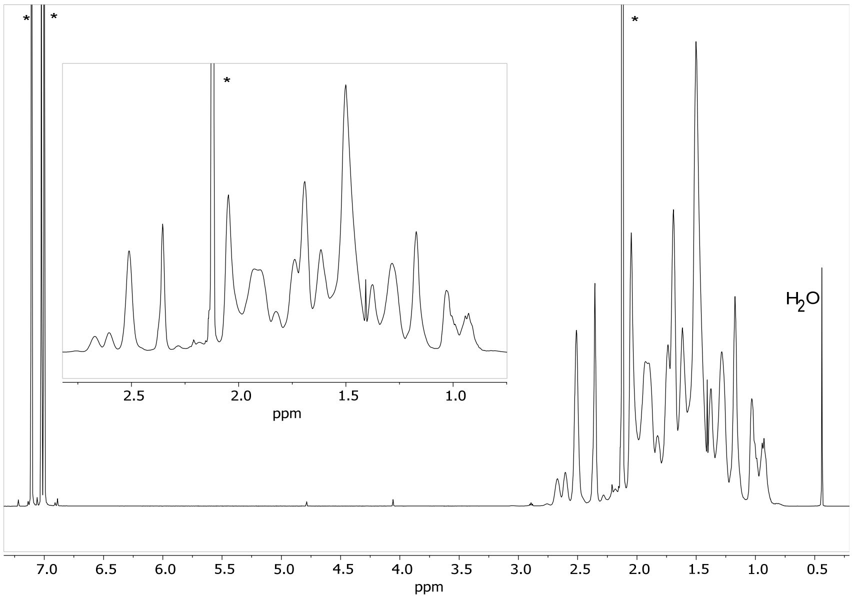

Figure S1: 700 MHz ^{1}H NMR spectrum of the COC-based substrate in toluene-d8 at 68 °C. * indicates solvent peaks.

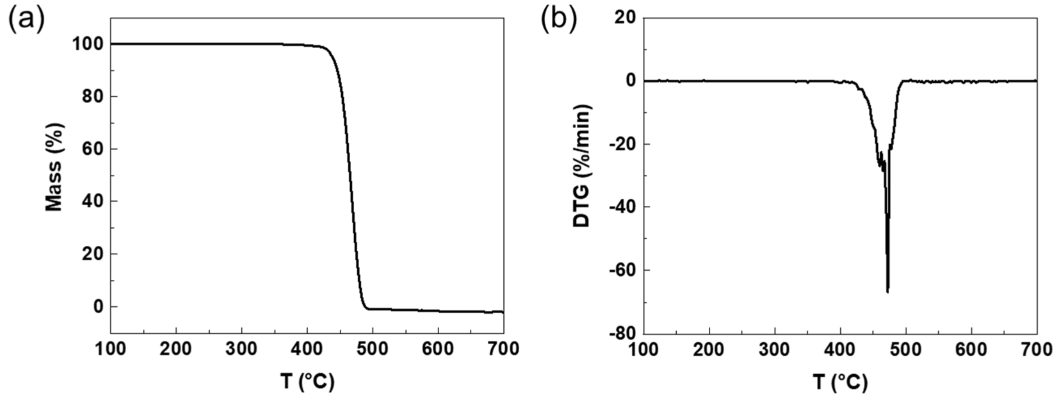

Figure S2: (a) TGA and (b) DTG curve of the COC-based substrate.

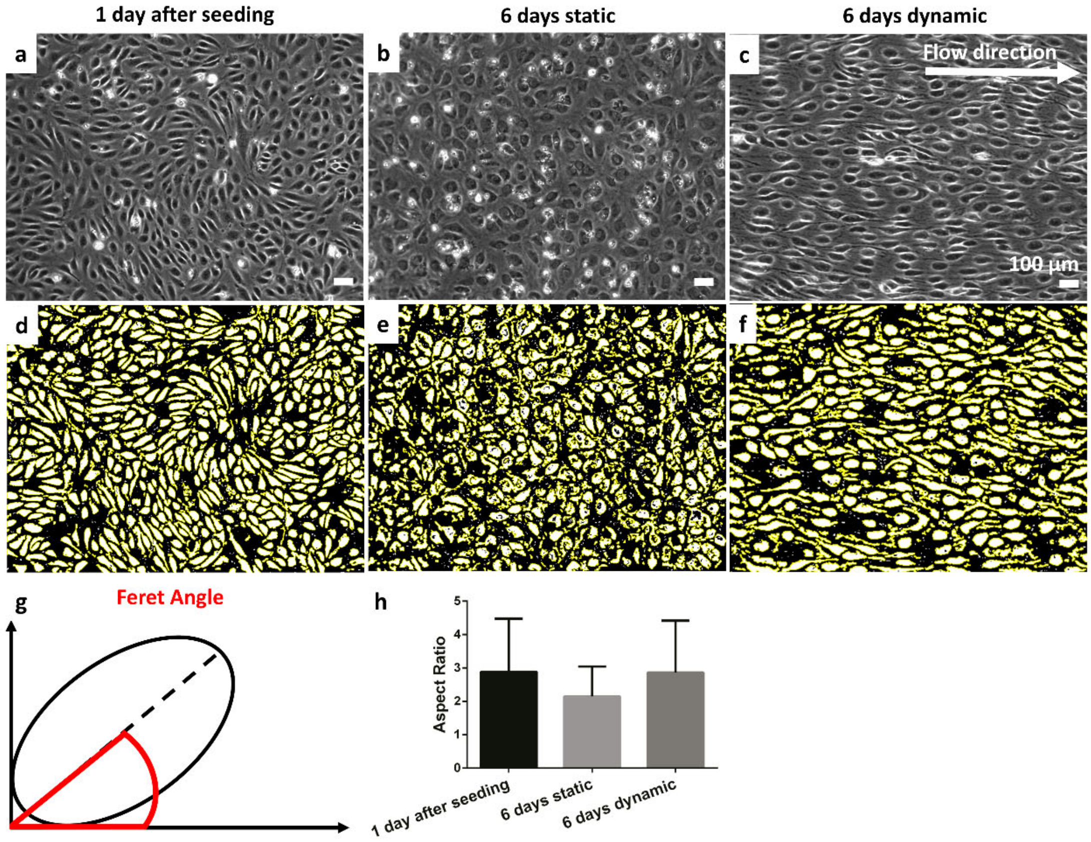

Figure S3: Quantification of endothelial cell (EC) alignment under static and dynamic culture conditions. EC were cultured for 1 day under static conditions (a) prior to the cultivation under quasi-static conditions (0.01 dyn · cm^{-2}, b) or laminar flow for six days (10 dyn · cm^{-2}, c). EC alignment was measured by the ImageJ software (d-f) and subsequent quantification of the Feret Angle (g). Feret Angle of statically cultured EC was greater than of EC cultivated under flow (h).
